# Changes in Metabolic Syndrome and Its Components Across the Pre-Pandemic, Pandemic, and Recovery Periods Among Korean Adults

**DOI:** 10.3390/jcm15197512

**Published:** 2026-09-27

**Authors:** Seong-Gil Kim, Do-Youn Lee

**Affiliations:** 1 Department of Physical Therapy, College of Health and Life Science, Korea National University of Transportation, Jeungpyeong-gun 27930, Republic of Korea; niceguygil@gmail.com; 2 College of General Education, Kookmin University, Seoul 02707, Republic of Korea

**Keywords:** metabolic syndrome, COVID-19, abdominal obesity, HDL-cholesterol, pandemic, epidemiology

## Abstract

**Background/Objectives:** Korean studies reported adverse metabolic changes during the COVID-19 pandemic, but subsequent patterns remain unclear. This study examined metabolic syndrome (MetS) and its components across pre-pandemic, pandemic, and recovery periods among Korean adults. **Methods:** We analyzed 36,194 adults aged ≥20 years from the 2018–2024 Korea National Health and Nutrition Examination Survey: 11,354 pre-pandemic (2018–2019), 10,200 pandemic (2020–2021), and 14,640 recovery (2022–2024). MetS was defined as ≥3 of five components. Complex-sample analyses and multivariable logistic regression were performed. Sensitivity analysis excluded reduced high-density lipoprotein cholesterol (HDL-C). **Results:** Weighted MetS prevalence was 29.1%, 30.5%, and 26.4% across the pre-pandemic, pandemic, and recovery periods, respectively. Individual MetS components showed divergent patterns. Abdominal obesity increased during the pandemic and remained elevated during recovery, whereas reduced HDL-C declined markedly during recovery. Compared with the pre-pandemic period, the fully adjusted odds of MetS were not significantly higher during the pandemic (OR, 1.077; 95% CI, 0.999–1.161) but were lower during recovery (OR, 0.845; 95% CI, 0.790–0.903). After excluding reduced HDL-C, the recovery association was attenuated (OR, 0.924; 95% CI, 0.857–0.997). **Conclusions:** MetS prevalence decreased during recovery, but individual components showed divergent trajectories, with abdominal obesity remaining elevated. Attenuation after excluding reduced HDL-C suggests that lower MetS prevalence should not be interpreted as uniform metabolic recovery.

## 1. Introduction

The COVID-19 pandemic was accompanied by widespread changes in daily activity patterns and health behaviors. Systematic evidence indicates that physical activity generally declined and sedentary time increased during pandemic-related restrictions across diverse populations [1]. Dietary behaviors also changed during the pandemic, although these changes were heterogeneous across populations, with studies documenting both favorable and unfavorable dietary shifts and, in some settings, increased consumption of unhealthy foods [2]. Such changes are relevant to metabolic health because metabolic syndrome (MetS) represents a clustering of central obesity, elevated blood pressure, dysglycemia, hypertriglyceridemia, and reduced high-density lipoprotein cholesterol (HDL-C) [3,4]. MetS is clinically important because it is associated with substantially increased risks of cardiovascular disease, cardiovascular mortality, and all-cause mortality [5]. Evaluating the recovery period is therefore important to determine whether metabolic changes observed during the pandemic persisted, reversed, or evolved differently after pandemic-related restrictions were relaxed.

Several Korean studies have reported adverse metabolic changes during the COVID-19 pandemic. Using KNHANES data, Kwon and Song observed an increasing prevalence of MetS through 2020, with particularly pronounced changes among middle-aged men [6]. Lee et al. similarly reported increases in obesity and several cardiometabolic conditions among Korean adults during the first pandemic year, particularly among men [7]. In the study most directly comparable to the present analysis, Chun et al. compared KNHANES 2018–2019 with 2020–2021 and reported an increase in MetS prevalence from 28.11% to 29.69%, accompanied by increases in elevated waist circumference from 31.31% to 36.59% and elevated fasting glucose from 36.42% to 39.41% [8]. Other Korean longitudinal and repeated cross-sectional studies have also reported deterioration in selected metabolic parameters, including abdominal obesity and glycemic profiles, during pandemic-related restrictions [9,10].

Most previous studies, however, focused on comparisons between pre-pandemic and pandemic periods [11,12]. Post-pandemic patterns therefore remain insufficiently understood. This distinction is particularly important because MetS is a composite phenotype, and a change in its overall prevalence may conceal substantially different trajectories among individual components [13,14]. Moreover, Korean secular-trend studies indicate that abdominal obesity and hyperglycemia had been increasing while reduced HDL-C had been decreasing over longer periods extending beyond the immediate pandemic comparison, suggesting that pandemic-related patterns may coexist with underlying population trends [11,12].

Therefore, using nationally representative KNHANES data from 2018 through 2024, this study investigated differences in the prevalence of MetS and its individual components across the pre-pandemic, pandemic, and recovery periods among Korean adults. We further examined the association between pandemic period and MetS after adjustment for demographic, socioeconomic, and health behavioral factors.

## 2. Materials and Methods

### 2.1. Study Population and Data Source

This study used data from the Korea National Health and Nutrition Examination Survey (KNHANES) conducted from 2018 through 2024. KNHANES is a nationally representative repeated cross-sectional surveillance program of the noninstitutionalized Korean population and uses a stratified, multistage probability sampling design. The survey includes health interviews, health examinations, and nutritional assessments.

A total of 50,742 individuals participated in KNHANES during the study period, including 16,102 participants in 2018–2019, 14,449 in 2020–2021, and 20,191 in 2022–2024. Participants aged <20 years were excluded (*n* = 8660). Among the remaining participants, 3134 were excluded because MetS status could not be determined owing to insufficient data on the required MetS components. A further 2754 participants were excluded because of missing covariate information. The final analytic sample therefore comprised 36,194 adults: 11,354 from the pre-pandemic period, 10,200 from the pandemic period, and 14,640 from the recovery period (Figure 1).

### 2.2. Definition of Pandemic Periods

Survey years were grouped into three population-level periods. The pre-pandemic period comprised KNHANES 2018 and 2019, the pandemic period comprised 2020 and 2021, and the recovery period comprised 2022 through 2024. The term recovery period refers to a population-level societal period following the major pandemic phase and relaxation of pandemic-related restrictions. The 2022–2024 survey years were grouped together to capture population-level metabolic patterns after the major pandemic phase.

### 2.3. Definition of Metabolic Syndrome

MetS was defined as the presence of at least three of five metabolic components, consistent with the harmonized conceptual framework for MetS, while using Korean-specific waist circumference thresholds [3,15]. The five components were abdominal obesity, defined as a waist circumference ≥ 90 cm in men or ≥85 cm in women; elevated blood pressure, defined as systolic blood pressure ≥ 130 mmHg, diastolic blood pressure ≥ 85 mmHg, or use of antihypertensive medication; elevated fasting glucose, defined as fasting glucose ≥ 100 mg/dL or use of oral glucose-lowering medication or insulin; elevated triglycerides, defined as a triglyceride concentration ≥ 150 mg/dL; and reduced HDL-C, defined as HDL-C < 40 mg/dL in men or <50 mg/dL in women. Because the analytical method used for triglyceride measurement in KNHANES changed in 2022, triglyceride values from 2018–2021 were harmonized to the analytical scale used from 2022 onward. For 2022–2024, the original triglyceride value was retained. This equation is consistent with a KNHANES method-comparison study demonstrating systematic differences between the glycerol-blanking method used through 2021 and the non-blanking method introduced in 2022 [16]. MetS component count was calculated only when all five components were available. Participants with zero to two components were classified as not having MetS, whereas those with three to five components were classified as having MetS.

A sensitivity analysis was conducted after excluding reduced HDL-C. The number of positive components among abdominal obesity, elevated blood pressure, elevated fasting glucose, and elevated triglycerides was calculated, and the sensitivity outcome was defined as the presence of at least three of these four non-HDL components. This analysis was performed because the laboratory responsible for HDL-C measurement in KNHANES changed in 2022, which may affect comparability across survey periods [17]. The analysis was intended to assess whether the primary findings remained consistent after excluding reduced HDL-C rather than to evaluate biological effect modification by HDL-C.

### 2.4. Sociodemographic and Health-Related Variables

Age was categorized as 20–29, 30–39, 40–49, 50–59, 60–69, and ≥70 years. Sex was classified as men or women. Educational attainment was categorized as elementary school or lower, middle school, high school, and university or higher. Marital status was classified as living with a spouse or not living with a spouse, with never-married participants included in the latter category. Personal income was classified into quartiles from Q1 (lowest) to Q4 (highest). Residential areas were classified as urban or rural.

Smoking status was categorized as current, former, or never smoking according to KNHANES smoking variables. Drinking status was classified as current drinking, defined as alcohol consumption at least once per month during the previous year, or non-drinking. Aerobic physical activity was classified according to the KNHANES aerobic physical activity indicator. Muscle-strengthening exercise was defined as participation in muscle-strengthening exercise on at least two days per week.

### 2.5. Statistical Analysis

All analyses were performed using IBM SPSS Statistics 30.0. The KNHANES complex sampling design was incorporated using the survey stratification variable, primary sampling unit, and the pooled sampling weight. This approach accounted for the stratified, clustered sampling design and unequal probabilities of selection. Categorical variables are presented as unweighted numbers and weighted percentages. General characteristics were compared according to MetS status within each pandemic period using complex-sample cross-tabulation analyses. Differences in the weighted prevalence of MetS and its individual components across the three periods were assessed using complex-sample procedures with survey-design-adjusted tests.

Complex-sample logistic regression was used to estimate odds ratios (ORs) and 95% confidence intervals (CIs) for MetS according to pandemic period, with the pre-pandemic period as the reference category. Four models were evaluated. The crude model included no adjustment variables. Model 1 was adjusted for age group and sex. Model 2 additionally included education, marital status, personal income, and urban/rural residence. Model 3 additionally included smoking status, drinking status, aerobic physical activity, and muscle-strengthening exercise. The same regression framework was applied to the sensitivity outcome defined as ≥3 of the four non-HDL MetS components. Tests for linear trend across the three periods were performed by entering pandemic period as an ordinal variable in complex-sample logistic regression. Statistical tests were two-sided, and *p* < 0.05 was considered statistically significant.

## 3. Results

### 3.1. General Characteristics of the Study Population According to MetS Status and Pandemic Period

The final analytic sample consisted of 36,194 adults, including 11,354 participants in the pre-pandemic period, 10,200 during the pandemic, and 14,640 during the recovery period. The numbers of participants with MetS were 3577, 3418, and 4118, respectively. Across all three periods, participants with MetS were more frequently represented in older age groups and were more often men. They were also more frequently represented in lower educational categories and rural areas. Differences according to MetS status were observed for marital status, personal income, smoking, drinking, and aerobic physical activity, although the patterns varied across periods (Table 1).

### 3.2. Prevalence of Metabolic Syndrome and Its Components Across Pandemic Periods

The weighted prevalence of MetS was 29.1% in the pre-pandemic period, increased slightly to 30.5% during the pandemic, and decreased to 26.4% during the recovery period (Table 2). Individual MetS components showed different temporal patterns. Abdominal obesity increased during the pandemic and remained elevated during recovery, whereas elevated fasting glucose returned toward the pre-pandemic level. Elevated triglycerides and reduced HDL-C decreased during recovery, while elevated blood pressure remained relatively stable across periods. When reduced HDL-C was excluded, the prevalence of having at least three of the four remaining MetS components did not differ significantly across periods, and no significant linear trend was observed (Table 2).

### 3.3. Association Between Pandemic Period and Metabolic Syndrome

Table 3 presents the associations between pandemic period and MetS. In fully adjusted Model 3, the pandemic period was not significantly associated with MetS compared with the pre-pandemic period (OR, 1.077; 95% CI, 0.999–1.161). The recovery period was associated with lower odds of MetS (OR, 0.845; 95% CI, 0.790–0.903). In the sensitivity analysis excluding reduced HDL-C, the pandemic period was not associated with the non-HDL outcome (OR, 1.042; 95% CI, 0.957–1.133). The recovery period remained associated with modestly lower odds (OR, 0.924; 95% CI, 0.857–0.997). Thus, the recovery-period association was attenuated after reduced HDL-C was excluded.

## 4. Discussion

In this nationally representative study of Korean adults, three principal findings emerged. First, weighted MetS prevalence was slightly higher during the pandemic than before the pandemic but was lower during the recovery period. After multivariable adjustment, the pandemic period was not significantly associated with MetS. In contrast, the recovery period was associated with lower odds than the pre-pandemic period. Second, the individual MetS components followed markedly heterogeneous trajectories. Abdominal obesity increased during the pandemic and remained elevated during recovery. Elevated fasting glucose approached its pre-pandemic level, whereas elevated triglycerides declined. Third, reduced HDL-C declined markedly during recovery and contributed substantially to the pattern observed for conventional MetS. When reduced HDL-C was excluded, both the prevalence difference and the adjusted recovery-period association were attenuated. Modestly lower adjusted odds, however, remained.

The pandemic-period findings are broadly consistent with previous Korean studies. Chun et al. reported that MetS prevalence increased from 28.11% in 2018–2019 to 29.69% in 2020–2021 and that the most pronounced component-level changes involved elevated waist circumference and fasting glucose [8]. Kwon and Song also reported a continuing increase in MetS prevalence through the initial pandemic period using KNHANES data, with larger changes in middle-aged men [6]. Other Korean studies identified increases in obesity or deterioration in several cardiometabolic indicators during pandemic restrictions [7,9,10]. The present study extends this literature by incorporating the 2022–2024 survey cycles and demonstrating that the metabolic pattern observed during the pandemic did not subsequently evolve uniformly across components.

The persistence of abdominal obesity is particularly noteworthy. Its weighted prevalence increased from 31.4% before the pandemic to 36.4% during the pandemic and remained at 35.7% during recovery. This pattern is compatible with previous KNHANES findings showing that abdominal obesity and glycemic abnormalities had already been major drivers of the long-term increase in MetS in Korea [12]. More recent analyses extending through 2022 similarly identified abdominal obesity and hyperglycemia as the components showing some of the largest long-term increases [11]. During the pandemic, lower levels of aerobic and transportation-related physical activity were also observed in Korean adults, and lower physical activity and greater sedentary behavior were associated with MetS [18,19]. These findings provide plausible context for the persistence of abdominal obesity. However, the repeated cross-sectional design cannot determine whether the same individuals gained or retained abdominal adiposity because of pandemic-related behavioral changes. The divergence between persistent abdominal obesity and lower overall MetS prevalence also shows why a decline in a composite diagnosis should not be interpreted as uniform metabolic recovery.

The results concerning reduced HDL-C require particularly cautious interpretation. The weighted prevalence of reduced HDL-C was essentially unchanged from the pre-pandemic to pandemic period (27.8% vs. 28.0%) but decreased to 18.8% during recovery. In parallel, the fully adjusted OR for conventional MetS during recovery was 0.845 (95% CI, 0.790–0.903), whereas the corresponding OR after excluding reduced HDL-C was attenuated to 0.924 (95% CI, 0.857–0.997). Moreover, the unadjusted weighted prevalence of the non-HDL sensitivity outcome did not differ significantly across periods (22.0%, 22.7%, and 21.1%; overall *p* = 0.096). These findings suggest that HDL-C-related changes contributed materially to the apparent decline in conventional MetS during recovery. The nonsignificant prevalence comparison and the modest association in the adjusted sensitivity model are not necessarily contradictory. The former represents an unadjusted population-level comparison. The latter estimates a conditional association after covariate adjustment.

At the same time, the reduction in reduced HDL-C cannot be assumed to represent either true biological improvement or a measurement artifact. Previous KNHANES laboratory research demonstrated that HDL-C measurements can be affected by assay calibration, with positive biases of approximately 2.85–9.40% documented in earlier survey cycles and year-specific calibration equations required to improve comparability with reference methods [17]. This historical evidence demonstrates that HDL-C surveillance estimates are sensitive to laboratory standardization. However, it does not establish that the marked reduction observed in 2022–2024 was caused by an assay discontinuity. Long-term KNHANES analyses have also documented a secular decline in the prevalence of low HDL-C [11,12]. Thus, at least part of the current pattern may reflect genuine population-level change. Both population-level changes and residual measurement heterogeneity should therefore be considered. The attenuation observed in the non-HDL sensitivity analysis indicates how strongly the conventional MetS result depends on the HDL-C component.

Several behavioral and healthcare-related factors may have contributed to the heterogeneous trajectories observed across periods. Systematic reviews have consistently shown substantial reductions in physical activity and increases in sedentary behavior during pandemic restrictions, whereas dietary changes were more heterogeneous and included both beneficial and unfavorable patterns. In Korea, KNHANES analyses showed lower-than-expected walking and aerobic physical activity during the first pandemic year. However, resistance exercise was higher, and total energy intake was lower, indicating that pandemic-related lifestyle changes were not uniformly adverse [19]. Routine chronic disease management also did not universally deteriorate. National data indicated broadly stable awareness, treatment, and control indicators for hypertension and diabetes during 2020, while several hypercholesterolemia management indicators improved [20]. The recovery-period pattern observed here may therefore reflect several factors, including changes in physical activity, diet, healthcare utilization and management, underlying secular trends, and population composition. It should not be attributed to a single pandemic-related mechanism.

From a clinical and public health perspective, these findings suggest that post-pandemic metabolic surveillance should extend beyond the binary presence or absence of MetS. MetS remains a useful summary phenotype because clustering of its components identifies individuals with elevated cardiovascular risk [5]. Nevertheless, a composite outcome may decline even when an important component remains persistently prevalent. In the present study, abdominal obesity represented such a residual burden during the recovery period. Monitoring the trajectories of waist circumference, glucose, blood pressure, triglycerides, and HDL-C separately may therefore provide a more informative assessment of population metabolic health than changes in MetS prevalence alone.

This study has several strengths. It used a large, nationally representative sample and incorporated KNHANES sampling weights, stratification, and clustering. The inclusion of seven survey years enabled comparison of pre-pandemic, pandemic, and recovery periods rather than restricting the analysis to a two-period comparison. Evaluation of individual MetS components provided information that would have been obscured by the composite outcome alone. The multivariable models accounted for major demographic, socioeconomic, and health behavioral factors, and the non-HDL sensitivity analysis provided additional insight into the dependence of the primary findings on reduced HDL-C.

Several limitations should be considered. First, KNHANES is a repeated cross-sectional survey, and different individuals were sampled each year. Therefore, individual longitudinal changes could not be evaluated, and the observational design precludes causal inference. Second, information on individual SARS-CoV-2 infection, disease severity, hospitalization, and post-COVID conditions was unavailable, and residual or unmeasured confounding may remain despite multivariable adjustment. Third, several health behavior variables, including smoking, drinking, and physical activity, were based on self-report and may be subject to reporting or recall bias. Fourth, the composition of KNHANES samples may have differed across survey periods despite the use of complex survey weights and statistical adjustment. In addition, the recovery period included three survey years, whereas the pre-pandemic and pandemic periods each included two, and this difference in duration may have affected comparisons across periods. The term recovery period represents an operational population-level societal period and should not be interpreted as clinical recovery at the individual level. Fifth, temporal comparisons involving reduced HDL-C require caution because laboratory-related differences may have affected comparability across survey cycles. Finally, ORs from the logistic regression models represent odds rather than prevalence ratios or risk ratios and should not be interpreted as direct measures of changes in population risk.

## 5. Conclusions

Among Korean adults, MetS prevalence was lower during the recovery period than before the pandemic, but individual components followed heterogeneous trajectories, with abdominal obesity remaining elevated. The attenuation observed after excluding reduced HDL-C indicates that the lower prevalence of conventional MetS should not be interpreted as uniform metabolic recovery. Continued population-level surveillance of individual metabolic components, particularly abdominal obesity, is warranted.

## Figures and Tables

**Figure 1:**
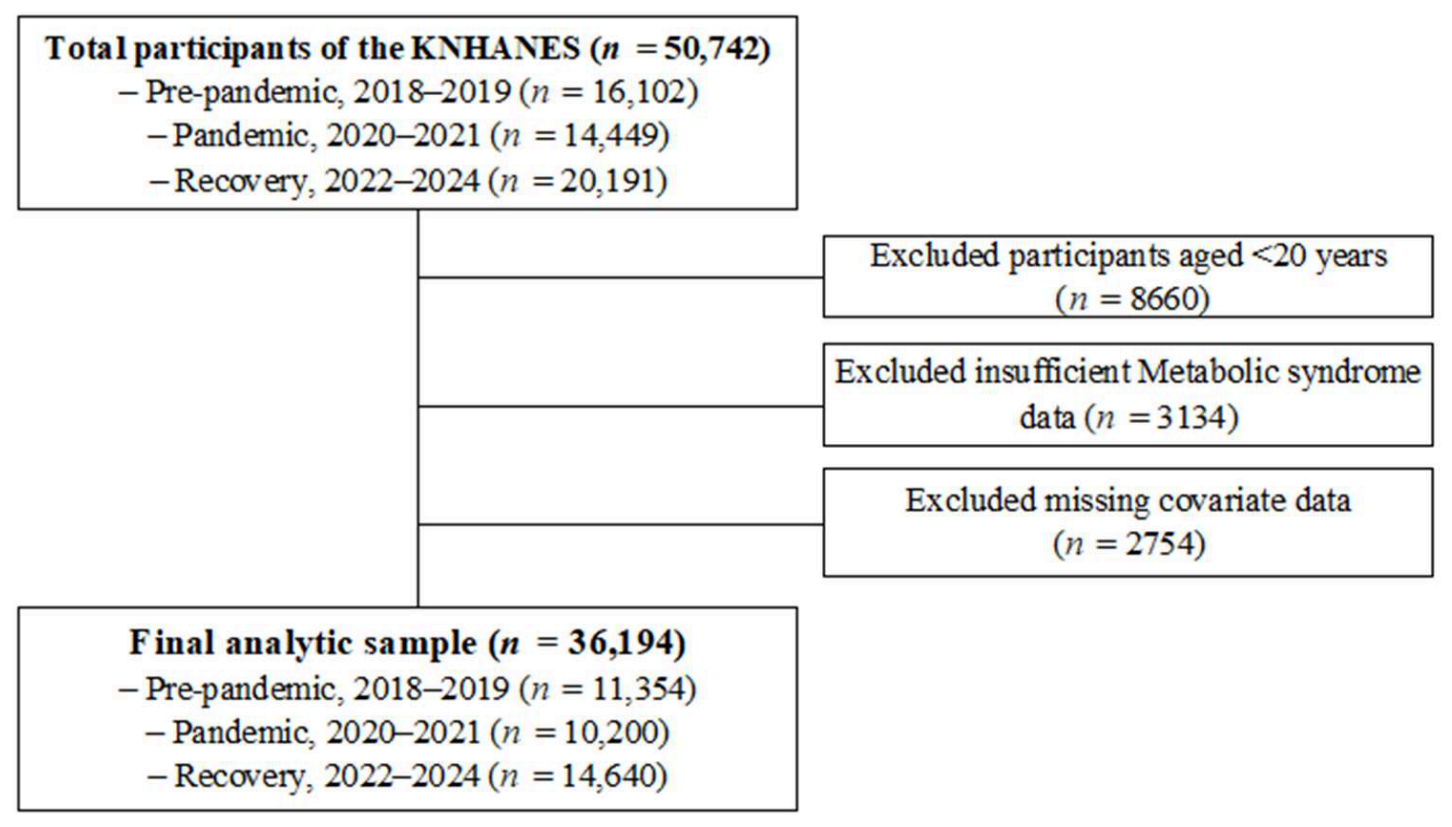
Flow chart of this study.

**Table 1:** General characteristics of the study population according to pandemic period and metabolic syndrome status.

Characteristic	Category	Pre-Pandemic		*p*	Pandemic		*p*	Recovery		*p*
		Non-MetS (*n* = 7777)	MetS (*n* = 3577)		Non-MetS (*n* = 6782)	MetS (*n* = 3418)		Non-MetS (*n* = 10,522)	MetS (*n* = 4118)	
		*n* (Weighted %)	*n* (Weighted %)		*n* (Weighted %)	*n* (Weighted %)		*n* (Weighted %)	*n* (Weighted %)	
Age	20 s	1216 (21.9)	104 (4.9)	<0.001	1154 (21.9)	129 (5.3)	<0.001	1517 (19.7)	108 (4.3)	<0.001
	30 s	1409 (20.2)	303 (11.6)		1044 (19.1)	253 (11.4)		1444 (17.7)	290 (11.2)	
	40 s	1571 (20.9)	540 (18.6)		1296 (20.2)	485 (18.5)		1872 (18.9)	520 (16.1)	
	50 s	1484 (18.6)	723 (24.1)		1241 (18.6)	682 (23.6)		1916 (18.4)	825 (23.6)	
	60 s	1151 (10.9)	902 (20.6)		1116 (12.5)	917 (22.6)		2104 (15.1)	1173 (23.9)	
	70 s+	946 (7.5)	1005 (20.2)		931 (7.7)	952 (18.5)		1669 (10.3)	1202 (20.8)	
Sex	Men	3210 (46.8)	1802 (57.6)	<0.001	2803 (46.1)	1773 (59.9)	<0.001	4125 (45.5)	2213 (62.3)	<0.001
	Women	4567 (53.2)	1775 (42.4)		3979 (53.9)	1645 (40.1)		6397 (54.5)	1905 (37.7)	
Education	Elementary or lower	876 (7.5)	954 (20.3)	<0.001	705 (6.4)	859 (17.4)	<0.001	1179 (7.2)	919 (15.8)	<0.001
	Middle school	650 (6.4)	509 (11.9)		553 (5.7)	489 (11.8)		898 (6.3)	604 (11.9)	
	High school	2128 (27.1)	1040 (31.0)		1876 (27.6)	966 (31.5)		2792 (25.4)	1242 (30.3)	
	University or higher	4123 (59.0)	1074 (36.7)		3648 (60.2)	1104 (39.3)		5653 (61.0)	1353 (42.0)	
Spouse	With	5281 (64.1)	2578 (72.6)	<0.001	4357 (61.2)	2372 (70.2)	<0.001	6898 (61.4)	2858 (68.1)	<0.001
	Without	2496 (35.9)	999 (27.4)		2425 (38.8)	1046 (29.8)		3624 (38.6)	1260 (31.9)	
Personal income	Q1 (Lowest)	1819 (23.9)	946 (26.6)	0.004	1541 (22.6)	916 (26.5)	0.005	2475 (23.2)	1103 (26.2)	0.002
	Q2	1924 (25.0)	914 (26.1)		1708 (25.1)	843 (24.5)		2603 (24.8)	1080 (25.8)	
	Q3	1974 (25.6)	900 (25.3)		1767 (26.1)	838 (25.0)		2668 (25.8)	1010 (24.6)	
	Q4 (Highest)	2060 (25.5)	817 (22.1)		1766 (26.2)	821 (24.1)		2776 (26.2)	925 (23.4)	
Region	Urban	6490 (86.9)	2753 (80.9)	<0.001	5532 (86.3)	2521 (80.5)	<0.001	8642 (86.1)	3060 (79.4)	<0.001
	Rural	1287 (13.1)	824 (19.1)		1250 (13.7)	897 (19.5)		1880 (13.9)	1058 (20.6)	
Smoking status	Current	1319 (20.0)	711 (23.3)	<0.001	1032 (17.2)	633 (22.4)	<0.001	1377 (15.1)	786 (22.8)	<0.001
	Past	1648 (21.2)	927 (26.8)		1451 (22.2)	963 (30.5)		2293 (22.9)	1184 (30.5)	
	Non	4810 (58.9)	1939 (49.9)		4299 (60.6)	1822 (47.1)		6852 (62.0)	2148 (46.7)	
Drinking status	Yes	4322 (59.2)	1838 (56.0)	0.011	3545 (56.3)	1612 (52.3)	0.001	5465 (55.5)	2053 (54.9)	0.575
	No	3455 (40.8)	1739 (44.0)		3237 (43.7)	1806 (47.7)		5057 (44.5)	2065 (45.1)	
Aerobic activity	Yes	3574 (48.7)	1248 (36.9)	<0.001	3041 (47.5)	1220 (38.2)	<0.001	5128 (52.1)	1488 (39.6)	<0.001
Resistance exercise	Yes	1806 (25.0)	604 (17.7)	<0.001	1740 (27.0)	625 (19.6)	<0.001	2833 (28.7)	806 (20.3)	<0.001

**Table 2:** Weighted prevalence of metabolic syndrome and its components according to pandemic period.

Outcome	Pre-Pandemic	Pandemic	Recovery	*p* Value	*p* for Trend
**MetS**	3577 (29.1%)	3418 (30.5%)	4118 (26.4%)	<0.001	<0.001
Sensitivity analysis (excluding reduced HDL-C)	2705 (22.0%)	2546 (22.7%)	3294 (21.1%)	0.096	0.21
**MetS components**					
Abdominal obesity	3746 (31.4%)	3916 (36.4%)	5331 (35.7%)	<0.001	<0.001
Elevated blood pressure	4855 (38.1%)	4315 (37.3%)	6276 (37.6%)	0.712	0.069
Elevated fasting glucose	4380 (35.6%)	4242 (38.2%)	5721 (36.0%)	0.007	0.615
Elevated triglycerides	3656 (32.6%)	3115 (31.6%)	3630 (26.4%)	<0.001	<0.001
Reduced HDL-C	3424 (27.8%)	3076 (28.0%)	2892 (18.8%)	<0.001	<0.001

**Table 3:** Odds ratios for metabolic syndrome according to pandemic period.

COVID-19 Period	Crude OR (95% CI)	Model 1 OR (95% CI)	Model 2 OR (95% CI)	Model 3 OR (95% CI)
**Met S**				
Pre-pandemic	Reference	Reference	Reference	Reference
Pandemic	1.070 (0.984–1.163)	1.055 (0.975–1.141)	1.073 (0.995–1.156)	1.077 (0.999–1.161)
Recovery	0.876 (0.814–0.942)	0.803 (0.748–0.861)	0.832 (0.778–0.889)	0.845 (0.790–0.903)
**Sensitivity analysis: ≥3 of 4 non-HDL MetS components**				
Pre-pandemic	Reference	Reference	Reference	Reference
Pandemic	1.042 (0.953–1.139)	1.023 (0.938–1.117)	1.038 (0.954–1.130)	1.042 (0.957–1.133)
Recovery	0.950 (0.878–1.028)	0.879 (0.813–0.951)	0.911 (0.844–0.983)	0.924 (0.857–0.997)

## Data Availability

Data was anonymized and can be downloaded from the website (https://knhanes.kdca.go.kr/knhanes, accessed on 2 June 2026).
